# Correction: Research into the thermal stability and mechanical properties of vitamin E diffusion modified irradiation cross-linked graphene oxide/ultra-high molecular weight polyethylene composites

**DOI:** 10.1039/d0ra90064h

**Published:** 2020-06-11

**Authors:** Weipeng Duan, Meiping Wu, Jitai Han

**Affiliations:** School of Mechanical Engineering, Jiangnan University Wuxi 214122 China wmp116699@163.com

## Abstract

Correction for ‘Research into the thermal stability and mechanical properties of vitamin E diffusion modified irradiation cross-linked graphene oxide/ultra-high molecular weight polyethylene composites’ by Weipeng Duan *et al.*, *RSC Adv.*, 2020, **10**, 4175–4188, DOI: 10.1039/C9RA09893C.

The authors regret the addition of an author, Zifeng Ni, to the original manuscript. The corrected author list is as shown above.

The authors regret that the funding information was incorrectly shown in the Acknowledgements section of the original manuscript. The corrected funding acknowledgement is as shown below.

This study is supported by the Additive Manufacturing Products Supervision and Inspection Center of Jiangsu Province, Wuxi Institution of Supervision & Testing on Product Quality, Wuxi, China.

The Royal Society of Chemistry apologises for these errors and any consequent inconvenience to authors and readers.

## Supplementary Material

